# Emerging roles of non-histone protein crotonylation in biomedicine

**DOI:** 10.1186/s13578-021-00616-2

**Published:** 2021-05-31

**Authors:** Jia-Yi Hou, Lan Zhou, Jia-Lei Li, De-Ping Wang, Ji-Min Cao

**Affiliations:** 1 Key Laboratory of Cellular Physiology At Shanxi Medical University, Ministry of Education, Key Laboratory of Cellular Physiology of Shanxi Province, and the Department of Physiology, Shanxi Medical University, Taiyuan, China; 2 Department of Clinical Laboratory, Shanxi Provincial Academy of Traditional Chinese Medicine, Taiyuan, China

**Keywords:** Lysine crotonylation, Post-translational modification, Protein, Cell biology, Disease

## Abstract

Crotonylation of proteins is a newly found type of post-translational modifications (PTMs) which occurs leadingly on the lysine residue, namely, lysine crotonylation (Kcr). Kcr is conserved and is regulated by a series of enzymes and co-enzymes including lysine crotonyltransferase (writer), lysine decrotonylase (eraser), certain YEATS proteins (reader), and crotonyl-coenzyme A (donor). Histone Kcr has been substantially studied since 2011, but the Kcr of non-histone proteins is just an emerging field since its finding in 2017. Recent advances in the identification and quantification of non-histone protein Kcr by mass spectrometry have increased our understanding of Kcr. In this review, we summarized the main proteomic characteristics of non-histone protein Kcr and discussed its biological functions, including gene transcription, DNA damage response, enzymes regulation, metabolic pathways, cell cycle, and localization of heterochromatin in cells. We further proposed the performance of non-histone protein Kcr in diseases and the prospect of Kcr manipulators as potential therapeutic candidates in the diseases.

## Introduction

Post-translational modifications (PTMs) of proteins make up most of the proteome and to a large extent, and establish the impressive level of functional diversity in higher multi-cellular organisms. Mounting evidence suggests that PTMs provide an elegant mechanism to govern protein function in diverse biological processes including cell differentiation and organismal development, and aberrant protein modification may contribute to diseases such as cancer. As the only amino acid with a side chain amine [1], lysine can be covalently modified by glycosyl [2], propionyl [3], butyryl [4], acetyl [5], hydroxyl [6], crotonyl [7], ubiquitinyl and ubiquitinyl-like [8], formyl [9], malonyl [10], succinyl [11], and methyl [12] groups. Some modifications, such as acetylation, methylation, and ubiquitination, have been extensively studied and are considered to be related to chromatin remodeling, gene transcriptional regulation, and other key biological processes.

Among the above PTMs, lysine crotonylation (Kcr) represents a newly discovered and often elusive PTM that has nonetheless the potential to alter the function of the modified protein. Histone crotonylation was first reported as a special marker of sex-related genes and was directly related to gene transcription [7]. Subsequently, some studies found that lysine crotonylation can play an important role in many diseases such as acute renal injury, depression, HIV latency, and cancer process by regulating the structure and function of histone [13–16]. In the past decade, mass spectrometry (MS)-based proteomics has greatly accelerated the discovery and identification of endogenous crotonylated proteins, and revealed the regulatory process of non-histone crotonylation. The discovery and identification of mammalian lysine crotonyltransferases (KCTs), lysine decrotonylases (KDCRs), and the crotonylation reader domain pave the way for the study of non-histone protein crotonylation. The identification of thousands of crotonylation sites has arisen great interest of biomedical researchers. More importantly, a growing number of studies have shown that non-histone protein crotonylation is involved in almost all major biological processes [17–21].

Here, we provide an overview of the expanding landscape of non-histone protein crotonylation, including the regulation, large-scale identification, biological function of protein crotonylation, and the crosstalk of Kcr with other PTMs. We further discuss the connection between protein crotonylation and diseases in which Kcr may be potential therapeutic targets of the diseases. Although there are not many reported in-depth investigations on the crotonylation of non-histone proteins, a comprehensive review on this rapidly developing field may help to uncover its biomedical significance and prospects.

### Uncovering lysine crotonylation

It is increasingly appreciated that combinations of PTMs can generate distinct protein isoforms with varying functions, which vastly expand the functional diversity of mammalian proteomes. The widespread occurrence of PTMs only started to become clear in the first years of the twenty-first century, when advances in high-resolution mass spectrometry enabled detection of thousands of low-abundance PTM sites. In this context, protein Kcr on histones was first described in 2011 by Zhao and colleagues [7], who designed a comprehensive method to systematically analyze histone PTM, using PTMap, an algorithm that can recognize all possible PTMs of proteins [22]. In this method, mass spectrometry (MS) analysis of histone hydrolyzed peptides maximizes sequence coverage and sensitivity, resulting in recognition of many new PTM sites, including Kcr identified as a new type of histone modification.

Histone crotonylation is an evolutionarily conserved histone post-translational modification appearing in eukaryotic cells from a wide range of species. Using a pan antibody against Kcr, Kcr signals in the core histones of sapiens (HeLa) cells, mouse, cerevisiae, elegans, melanogaster, as well as plant, have been detected [7, 23–26]. The modified proteins and sites vary and overlap among different species, such as H2AK125cr in humans and mice [26].

These groundbreaking discoveries set the stage for the field of non-histone protein crotonylation. In 2017, Xu et al. [17] first reported that in addition to histones, Kcr also occurs on a variety of non-histone proteins. Increasing proteomic analyses have demonstrated that the frequency of non-histone protein crotonylation is very high, and these proteins are involved in many key cellular processes which are related to physiology and disease in eukaryotic cells. These mass spectrometry-based studies have quantified the relative effects of tens of thousands of crotonylation sites on genetic and metabolic activities, and provided insight into the dynamic regulation of Kcr.

### Regulation of crotonylation

**Fig. 1:**
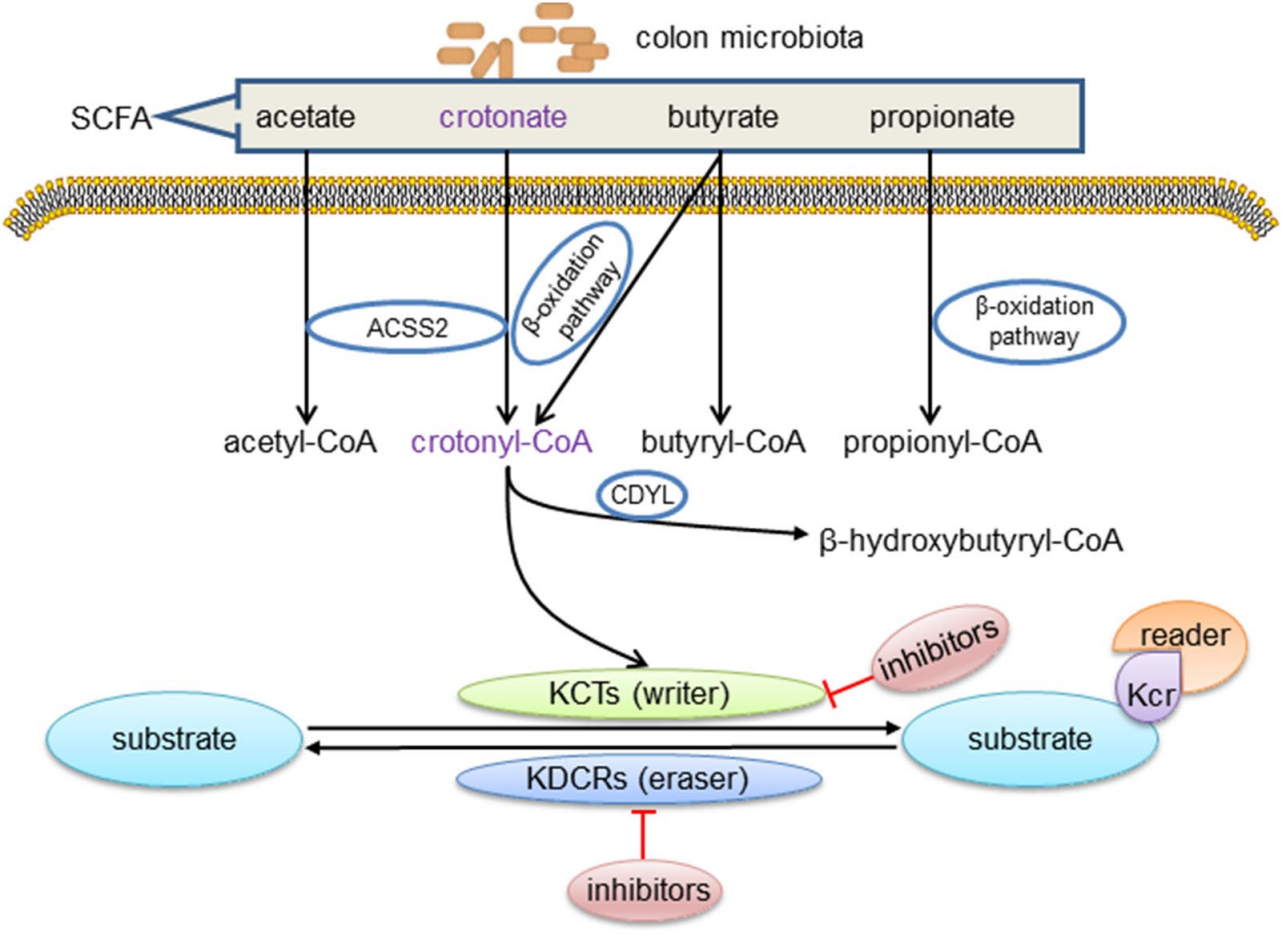
The metabolic pathways and enzymes and modulators for lysine crotonylation (Kcr). Colon microbiota is a source of short chain fatty acids (SCFAs) which may be metabolized to acetyl/crotonyl/propionyl/butyryl-CoA in host cells. These CoAs are precursors used by enzymes to promote lysine acetylation (Kac), crotonylation (Kcr), propionylation, and butyrylation of histone and non-histone proteins. Currently characterized crotonyltransferases (KCTs) (writer) include CBP/P300, MYST, and GNAT family, and histone decrotonylases (KDCRs) (eraser) include HDAC I and HDAC III. When substrates are histone proteins, Kcr readers, including bromodomains, YEATS, and DPF domain, recognize the Kcr. Chromodomain Y-like (CDYL), acting as a crotonyl-CoA hydratase which converts crotonyl-CoA to β-hydroxybutyryl-CoA, negatively regulates crotonylation. ACCS2, acyl-CoA synthetase short chain family member 2. Inhibitors of P300/CBP include C646 and SGC-CBP30. Inhibitors of HDACs include trichostatin A (TSA), nicotinamide (NAM), suberoylanilide hydroxamic acid (SAHA), and LBH589

There are structural similarities between lysine crotonylation and lysine acetylation, and in fact, many of the “modulators” of crotonylation are the same as those of acetylation [27, 28]. However, crotonylation is unique in its π-electron conjugation, resulting in being rigid and planar in shape. Crotonyllysine but not acetyllysine is found to more robustly neutralize the positive charge because of a longer carbon chain [29]. Histone crotonylation have been found sharing the similar set of regulatory machineries as histone acetylation [19, 30]. In the process of crotonylation, crotonyl is added to the substrate proteins by lysine crotonyltransferase (KCT) (“writer”) [17, 31–33] and then histone crotonylation can be recognized by another family of proteins such as YEATS2, AF9, and Taf14 (“reader”) [29, 34, 35], which usually initiates a series of downstream signals [29]. At the end of PTM-induced signal transduction, most crotonylation can also be removed by lysine decrotonylase (KDCR) (“eraser”) [17, 36–38]. Part of the effect of either KCT or KDCR can be selectively counteracted by drug inhibitors (Fig. 1). The crotonyltranferase, decrotonylase, reader, and donor of non-histone and histone crotonylation do not completely overlap, which are respectively described below.

#### Lysine crotonyltranferase (writer), decrotonylase (eraser), and reader

The modulators of histone crotonylation have been extensively studied and summarized earlier [19, 39], and some of them can also regulate non-histone crotonylation. Six KCTs have been identified in metazoans and are classified into three major families: P300/CBP (p300/CREB-binding protein) [40], MYST (Moz, Ybf2, Sas2, and Tip60) [32], and GNAT (GCN5-related N-acetyltransferase) [33]. So far, p300 and hMOF in MYST family and PCAF in GNAT family were revealed to be non-histone crotonyltransferase (Table 1). P300 and its homologue CBP are two highly related KCTs that function as general transcriptional coactivators. The human males absent on the first (hMOF) protein which belongs to the MYST family, and the P300/CBP-associated factor (PCAF) which is a member of the GNAT family, both possess evolutionarily conserved KCT activity [17, 32]. CBP, p300, and hMOF have the same enzyme reaction centers which catalyze acetylation and crotonylation at multiple sites. It is found that the activity of each KCT varies from substrate to substrate (Table 1). The non-histone NPM1 can be strongly crotonylated by CBP and hMOF, but only moderately crotonylated by PCAF. However, crotonylation of DDX5 can be catalyzed by CBP, but not by other KCTs [17].

**Table 1:** Lysine crotonyltranferases and their histone and non-histone substrates

Family	Enzyme name	Substrates and modified lysine residues			
		Histone	References	Non-histone	References
p300/CBP family	p300, CBP	H3K18	[31]	NPM1, DDX5	[17]
MYST family	hMOF	H3K4, H3K9, H3K18, H3K23, H4K8, and H4K12	[32]	NPM1	[17]
	Esa1	H4K5, H4K8, H4K12, and H4K16	[33]	N/A	N/A
GNAT family	GCN5	H3K9, K3K14, and H3K18A	[33]	N/A	N/A
	PCAF	N/A	N/A	NPM1	[17]

*DDX5* DEAD-box helicase 5, *Esa1* essential Sas2-related acetyltransferase 1, *GCN5* general control nonrepressed-protein 5, *GNAT* GCN5-related N-acetyltransferase, *hMOF* human males absent on the first, *MYST* Moz, Ybf2, Sas2, and Tip60, *NPM1* nucleophosmin-1, *N/A* not available, *PCAF* P300/CBP-associated factor, *P300/CBP* P300/CREB-binding protein

Most lysine decrotonylases have a wide range of substrates, including histone and non-histone proteins. KDCRs can be grouped into two major categories: Zn^2+^-dependent KDCRs and NAD^+^-dependent sirtuin decrotonylases (Table 2). The Zn^2+^-dependent KDCRs are often referred to as classical HDACs and belong to class I [41]. In class I HDACs, HDAC1 and HDAC3, but not HDAC2, can decrotonylase non-histone NPM1, and this effect can be reversed by treatment with HDACs inhibitors such as trichostatin A (TSA). Similarly, two other HDAC inhibitors, suberoylanilide hydroxamic acid (SAHA) and LBH589, can promote the crotonylation of NPM1 [17] (Fig. 1). Sirtuin family deacetylases (SIRTs), which are also referred to as class III HDACs, localize to different cellular compartments, including the nucleus (SIRT1), cytoplasm (SIRT2), and mitochondria (SIRT3) [42, 43]. So far, it has not been reported that Sirtuin family can decrotonylase non-histone protein. In the function and regulation of protein crotonylation, Kcr must be recognized by certain proteins possessing special structures, these proteins are called *reader*, including bromodomain, double plant homeodomain (PHD) finger (DPF), and YEATS domains [29, 34, 35, 44–46]. Reader originally refers to a chromatin binding protein module that recognizes the covalent modification of histone. At present, there is no evidence that non-histone KCR also has a reader.

**Table 2:** Different classes of lysine decrotonylases, their co-factors, cellular locations, and substrates

Co-factor	Class	Members	Location	Histone	Substrate		
					References	Non-histone	References
Zn^2+^-dependent	HDAC I	HDAC1	Nucleus	H3K4, H3K9, H3K18, H3K23, H4K8, and H4K12	[36]	NPM1	[17]
		HDAC2	Nucleus	H3K9, H3K18, and H4K8	[36]	N/A	N/A
		HDAC3	Nucleus	H3K9, H3K18, and H4K8	[36]	NPM1	[17]
		HDAC8	Nucleus	H3K4, H3K9, H3K18, H4K8, and H4K12	[36]	N/A	N/A
NAD^+^-dependent	HDAC III	SIRT1	Nucleus/ cytoplasm	H3K4, H3K9, and H4K8	[36–38]	N/A	N/A
		SIRT2	cytoplasm	H3K4 and H3K9	[37, 38]	N/A	N/A
		SIRT3	Mitochondria	H3K4	[38]	N/A	N/A

*N/A* not available

#### Crotonyl-CoA (donor)

Crotonyl-CoA is presumed a crotonyl donor in the process of Kcr. Crotonate, mainly produced by the colon microbiota, is the short-chain fatty acid (SCFA) precursor of crotonyl-CoA [47]. Circulating short-chain fatty acids (SCFA), such as acetate, crotonate, butyrate, and propionate, are taken up by tissues and converted to acyl-CoA by the acyl-CoA synthetase short chain family member 2 (ACSS2) or eventually yield crotonyl-CoA through different metabolic pathways such as fatty acid β-oxidation pathway and lysine degradation [48] (Fig. 1). Intracellular and intercellular metabolism can affect Kcr by changing the concentration of crotonyl-CoA in the following two ways:

First, direct elevation of crotonyl-CoA by sodium crotonate. Sodium crotonate (NaCr) enhances non-histone crotonylation most likely through conversion of NaCr to crotonyl-CoA. Adding NaCr to the cell culture media can lead to a significant increase in the crotonylation of many non-histone proteins, including ubiquitin ligase RNF2 and the binding enzymes UBE2E1, NCOR1, and RBBP4, which are components of histone deacetylase complex 12 [49]. Crotonylation of proteins induced by NaCr may also be catalyzed by enzymes. NaCr can specifically enhance protein crotonylation but not acetylation in wild HeLa cells, and this induction can be nearly completely eliminated by C646 and SGC-CBP30, two selective P300/CBP inhibitors [49].

Second, enzymatic regulation of crotonyl-CoA level. The enzymes involved in the conversion of crotonate to crotonyl-CoA can affect the crotonylation. Chromodomain Y-like (CDYL) protein, which contains an N-terminal chromodomain and a C-terminal enoyl-coenzyme A hydratase/isomerase catalytic domain (also known as CoA pocket or CoAP), has been implicated in epigenetic regulation and transcription repression by targeting chromatin through its N-terminal chromodomain [50]. Liu et al. [51] reported that CDYL, acting as a crotonyl-CoA hydratase, can add a water molecule to the double bond at the position between the second and third carbon, and negatively regulate histone Kcr (Fig. 1). Yu et al. [52] reported that CDYL can negatively regulate the Kcr of RPA1 on multiple lysine sites, which is a reaction to DNA-damaging insults.

### Large-scale identification of non-histone protein crotonylation

The breakthroughs in high-resolution mass spectrometry (MS)-based proteomics have enabled both the identification of thousands of crotonylation sites and the relative quantification of these sites in a single experiment, ushering in the age of crotonylomics (proteome-wide characteristics of crotonylation). Crotonylomics mapping is a prerequisite for systems-level analyses and provides an important resource for discovering novel properties and regulatory functions of crotonylation.

#### Quantitative mass spectrometry for crotonylomics analysis

**Fig. 2:**
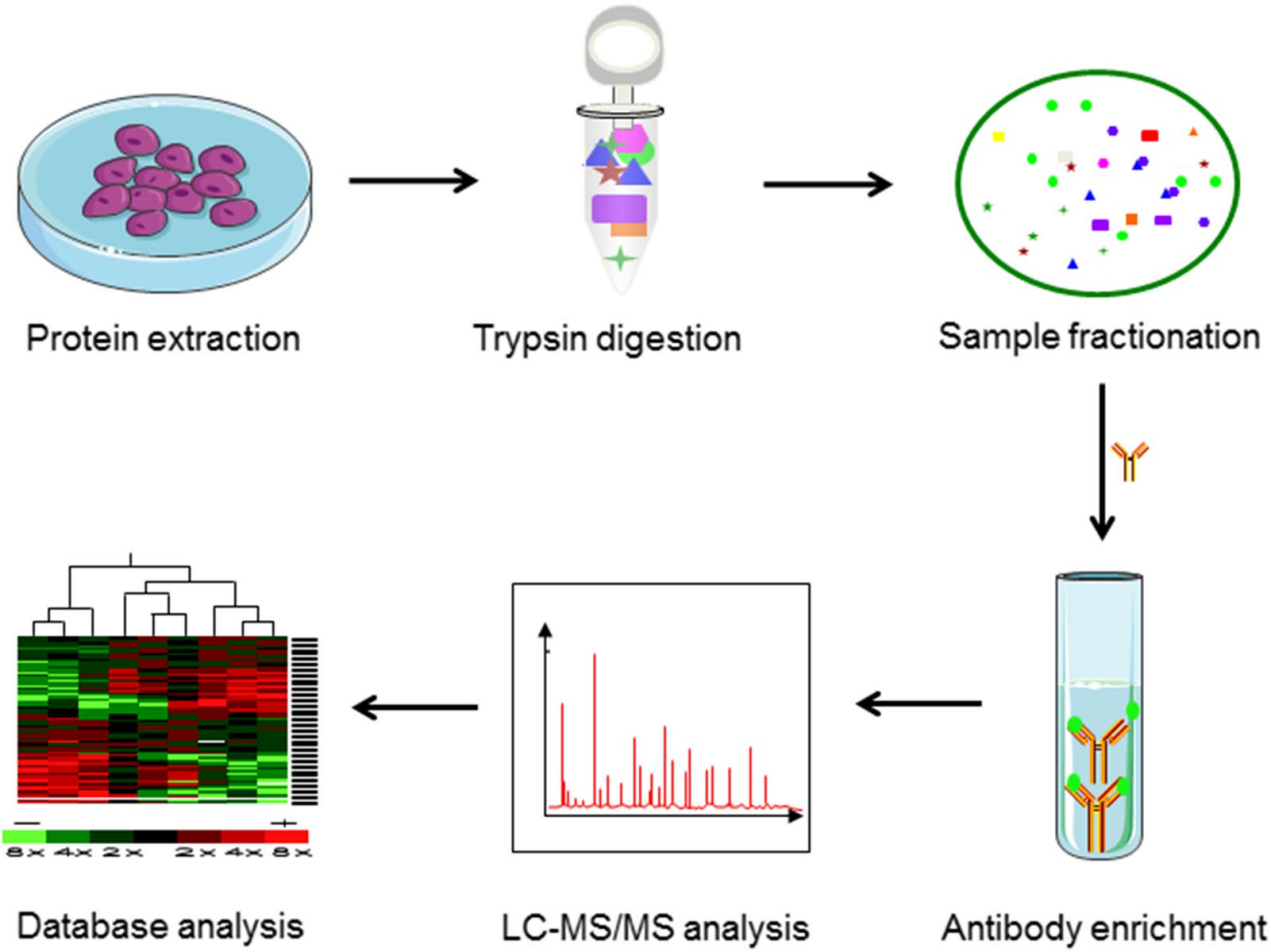
Schematic diagram of the experimental procedures for mass spectrometry-based global analysis. Proteins are extracted from cells or tissues and digested into peptides with a protease such as trypsin. The tryptic peptides are then separated and fractionated by high pH reverse-phase high performance liquid chromatography (HPLC). Proteolysis of whole-cell protein extracts generates numerous peptides, but only a small fraction is crotonylated. To enrich lysine crotonylated peptides, pan-Kcr antibodies are applied to identify the crotonylated peptides in complex peptide mixtures using immunoaffinity purification. The resulting peptides are ionized in the electrospray source before entering the mass spectrometer. MS and MS/MS spectra are then computationally processed to deduce peptide sequences, including the presence and location of crotonylation, and to quantify the abundance of crotonylated peptides and proteins

Various MS instruments and methodological approaches can be used to perform crotonylomics analysis for proteins and peptides. Nearly all large-scale crotonylation studies use the “shotgun” or bottom‑up proteomics approach, which involves enzymatic digestion of all proteins (the proteome) followed by liquid chromatography coupled to tandem MS (LC–MS/MS) (Fig. 2).

Crotonylation of some protein sites cannot be detected in wild types cells. In order to find out which lysine sites may be crotonylated, some studies use drugs that promote crotonylation or knock down the expression of “modulators” of crotonylation during cell culture, such as sodium crotonate (NACR) which promotes non-histone crotonylation by conversion to crotonyl-coenzyme A [49], and HDAC inhibitor SAHA [21] or crotonyl-CoA hydrolase CDYL [52] which negatively regulate Kcr. But more researchers collect biological samples under interested conditions for modification (Table 3).

**Table 3:** Global profiling of non-histone crotonylation based on LC–MS/MS analysis

Biological samples analyzed	Experiment condition	Identified proteins	Kcr sites	Amino acids flanking the identified Kcr sites	Subcellular localization	Biological function	Validated proteins by WB	Year	References
H. sapiens									
IgAN patients	PBMCs were obtained and compared with normal controls	353	770	Alanine (A), aspartic acid (D), and lysine (K) residues at the − 1 to + 1 positions	Organelles, extracellular regions and membranes (69%)	Upregulated proteins involved: focal adhesion, cell-substrate junction, cell-substrate adherens junction, biological process of regulation of body fluid levels, and calcium ion binding.Downregulated proteins involved: cellular component of blood microparticle, biological process of humoral immune response, and ubiquitin-like protein ligase binding	N/A	2020	[68]
HeLa cells	CDYL KO	3734	14,311	Negatively charged glutamic acid (E) at − 1 and + 1 positions	Mitochondria (18%), cytoplasm and nucleus (roughly equal)	Translation initiation, RNA splicing, and amino acid metabolism	RPA1, APEX1, and HP1	2020	[52]
HCT116 cells	P300 KO	392	816	Glutamic acid (E) at − 4, − 1, + 1, and + 4 positions, valine(V) at + 2 position	Nucleus (69%), cytosol (89%), and mitochondria (19%)	Co-translational protein targeting to membrane, nuclear-transcribed mRNA catabolic process, and translational initiation	LMNB2	2018	[61]
PBMCs of patients with hemodialysis maintenance	PBMCs were obtained and compared with normal controls	347	1109	Aspartic acid (D) and glutamine (E) at − 1 to + 4 position; lysine (K) at − 10 to − 5 and + 10 to + 5 positions	Upregulated crotonylated proteins: cytoplasm (46%), nucleus (14%), mitochondria (12%), and extracellular region (11%). Downregulated proteins: cytoplasm (57%), extracellular region (11%), mitochondria (9%), and nucleus (8%)	Upregulated proteins enriched in cellular metabolic process and cellular response to outside and downregulated proteins localized in cellular functions such as migration and development	N/A	2018	[56]
H1299 cells	Without any treatment	1024	2696	Glutamate (E) residues at the − 1 and + 1 positions	Cytoplasm (40%), nucleus (27%), and mitochondria (13%)	18% of crotonylated proteins are involved in the cell process, 14% are involved in metabolism, 13% are single biological proteins, and 11% are biomodulatory proteins	NPM1, FHL1, ACTN1, Integrin β1, Vinculin, ERK2, CDK1, GAPDH, and OTUB1	2017	[17]
HeLa cells	Treated with NaCr	453	558	Alanine (A) residues at + 3 or − 6 positions	Nuclear proteins (62.3%), nucleoplasmic localization proteins (9.4%), other cellular components including plasma membrane, cytoplasm, Golgi apparatus, and unclassified proteins (28.3%)	RNA processing, nucleic acid metabolism, chromosome organization, gene expression, DNA conformational change and packaging, chromatin organization and cell cycle	CBX3, CBX5、MTA2, Cul4B, and HDAC1	2017	[49]
	Without any treatment	70	N/A	N/A	N/A	Various molecular functions and biological processes closely related to DNA and RNA metabolism and cell cycle	HDAC1	2017	[49]
A549 cells	Treated with SAHA	2021	8475	N/A	Cytoplasm (35.7%), nucleus (27.4%) and mitochondria (13.5%)	Gene expression, transcription, protein folding, and a variety of metabolic processes	N/A	2017	[21]
Others									
*T. gondii*	RH strain	3735	1,061	Isoleucine (I) and lysine (K) occurred upstream; leucine (L), lysine (K) and phenylalanine (F) occurred downstream	Concentrated in and around the nucleus	Related to protein translation, compound metabolism, and biosynthesis	N/A	2019	[71]
	ME49 strain	3396	984						
The tea plants	NH4 + deficiency/resupply	971	2288	Alanine (A), glutamate (E), and lysine (K) residues at surrounding positions	Chloroplast (36.8%), cytoplasm (33.0%), and nucleus (13.7%)	Photosynthesis, carbon fixation, and amino acid metabolism	N/A	2019	[60]
*Oryza sativa L. japonica*	Without any treatment	690	1265	Aspartic acid (D), glutamine (E), isoleucine (I), lysine (K), leucine (L), arginine (R), and valine (V) at surrounding positions	Chloroplast (51%), cytoskeleton (1%), endoplasmic reticulum (1%), and extracellularly located (2%)	Photosynthesis	N/A	2018	[70]
*Carica papaya L.*	Without any treatment	2120	5995	Alanine (A), aspartic acid (D), and glutamine (E) at − 5 to − 1 and + 1 to + 5 positions, while lysine(K) and arginine (R) at − 10 to − 5 and + 5 to + 10 positions	Chloroplast (713 peptides), cytosol (691 peptides), nucleus (290 peptides), and mitochondria (138 peptides)	Biosynthesis of antibiotics, carbon metabolism, biosynthesis of amino acids, and glycolysis	N/A	2018	[69]
*Zebrafish embryos*	Without any treatment	218	557	Hydrophobic (L, I, V) and acidic (D and E) amino acids at surrounding positions	Cytosol (58%), mitochondria (11%), extracellular matrix (8%), and plasma membrane (6%)	Translation, metabolic process, and diverse regulation of skeletal muscle contraction	N/A	2018	[18]
PAT	Degradation in Rhodotorula mucilaginosa	629	1691	Negatively charged residues from − 10 to + 10	Mitochondria (39%), cytosol (32%), and nucleus (14%)	Metabolic process, cellular process, and single-organism process with upregulated or downregulated proteins	N/A	2018	[54]
* Nicotiana tabacum*	Without any treatment	637	2044	Aspartic acid (D) and glutamine (E), at surrounding positions	Chloroplast (37%), cytosol (30%), nucleus (12%), and mitochondria (5%)	Carbon metabolism, the citrate cycle, glycolysis, and photosynthesis the biosynthesis of amino acids	N/A	2017	[25]

*A549* human lung adenocarcinoma cells, *CDYL* chromodomain Y-like protein, *HCT116* human colon cancer cells, *HeLa* human cervical adenocarcinoma cells, *H1299* human lung adenocarcinoma cells, *IgAN* immunoglobulin A nephropathy, *KO* knockout, *NaCr* sodium crotonate *N/A* not available, *PAT* patulin, *PBMCs* peripheral blood mononuclear cells, *SAHA* suberoylanilide hydroxamic acid, *T. gondii* toxoplasma gondii parasites

In the next workflow, proteins are extracted from biological samples and digested into large numbers of peptides by enzymatic hydrolysis (usually trypsin), only a small part of the peptides is crotonylated. In order to identify the modified sites under specific conditions, quantitative analysis should be used. The most commonly used methods are to identify modified sites by comparing the intensity of crotonylated peptides among different samples, including metabolic labeling, chemical labeling, and label-free quantification. Each method has its advantages and disadvantages. SILAC (stable isotope labelling by amino acids in cell culture), which belongs to metabolic labeling, is one of the most commonly used methods in quantitative proteomics [53]. Natural isotopes (light) or stable isotopes (medium/heavy) are used to replace the corresponding amino acids during cell culture. Its advantage is the high efficiency for protein labeling, while its disadvantage is time consuming. Metabolic labeling has been successfully used to reveal the relative changes of crotonylation of non-histone proteins in homologous recombination-mediated DNA repair [52].

Chemical labeling can be used in samples that cannot be metabolically labeled, and several samples can be quantified in parallel, for example, by tandem mass tags (TMTs) [54] or by isobaric tags for relative and absolute quantification (iTRAQ) [55, 56]. However, due to the influence of labeled groups, the identification flux was lower than the label-free approach.

Label-free quantification allows direct identification and quantification of proteins in a large scale, usually requiring analysis of a sample in triplicate to ensure that the measured differences are statistically significant [17, 20]. However, the accuracy of label-free quantification is slightly worse than that of the labeled quantification, because the former may be affected by the stability of mass spectrometry and other factors. In recent years, mass spectrometry techniques with increased stability and repeatability, as well as greatly improved computational algorithms for quantification of MS data, have made label-free quantification an attractive option [57–59].

To reduce the complexity of samples, after digestion, the trypsin polypeptides can be divided into several components by high-pH reversed-phase fractionation (RPF), known as sample fractionation. To increase the depth of the crotonylation analysis, immunoaffinity purification is usually used, in which the pan-Kcr antibody is immobilized to a resin bead and selectively bound to the crotonylated tryptic peptides and is then eluted [17, 52, 60, 61]. Enrichment of crotonylated peptides is usually combined with sample fractionation to improve the efficiency of the next mass spectrometry (MS). Finally, the enriched peptides are usually separated by liquid chromatography and ionized in the electrospray source, and entered into the mass spectrometer for analysis. High-resolution and high-quality precision analyzers can detect hundreds or thousands of different molecular features in a single LC–MS experiment, but only a small fraction of which can be identified and quantified [62]. The abundance of these eluted peptides, which range over many orders of magnitude, is a formidable analytical challenge that has been driving the progress of faster and more sensitive instruments and detection modes over the past decades [63–65]. For instance, a scan mode termed parallel accumulation-serial fragmentation (PASEF) has recently been demonstrated to increase sequencing speed exponentially without loss of sensitivity [66, 67]. All these technological advances are high performance additions to the technology toolbox in crotonylomics.

Other experiments can also be used to verify the results of crotonylomics, such as western blotting and immunofluorescent staining. Immunofluorescent staining shows that crotonylated proteins are widely located in the cytoplasm and nuclei of H1299 and HeLa cells [17]. In addition, crotonylated proteins are widely found in a variety of tissues of mice, including lung, kidney, liver, colon, uterus, and ovary. Although these methods are not as efficient as MS, they can be used to verify the conclusions of large-scale experiments, such as NPM1, FHL1, ACTN1, integrin β1, ERK2, and CDK1, which are considered to be crotonylated as assayed by MS and can also be detected by western blotting [17].

#### Proteomic characteristics of crotonylation

Due to the progress of technology, the field of lysine crotonylation has developed rapidly in the past few years. Crotonylation was initially found to occur mainly in histones. However, a significant conclusion from recent proteomic studies is that most crotonylation events occur on non-histone proteins [17, 52, 56, 61, 68]. MS-based proteomics methods are now used in a variety of organisms not limited to humans, resulting in the identification of thousands of new crotonylation sites (Table 3). These studies show that crotonylation sites are often conserved in different organisms [18, 60, 69–71], thus it is obvious that crotonylation can be regarded as a protein modification that exists prevalently in all fields of life. Crotonylated non-histone proteins are widely distributed in subcellular compartments and participate in a variety of important cellular functions, signal pathways, and variant biological activities (Table 3).

Motifs refer to some specific amino acids sequences which localize near the lysine acylation site and are generally highly conserved. The identification of the sequence of modification sites and the study of the corresponding model peptides provides clues for predicting the potential modification sites of new proteins. For instance, motif analysis is often used to predict potential kinase phosphorylation sites in bioinformatics [68, 72]. The amino acid sequences of motifs have been extracted from the upstream and downstream of the crotonylated lysine residue sites, which can describe the sequence commonness around the crotonylation sites. The studies given in Table 3 describe the amino acid sequence background of the crotonylation sites in eukaryotes. The highly conserved amino acids in these motifs, that is, E and D [17, 25, 52, 56], are negatively charged [54], which may represent the specific amino acid bias near the histone and non-histone crotonylation sites. Although specific amino acids are preferred near crotonylated lysine, no clear crotonylated amino acid sequence is found, which may be due to the diversity of KCT which targets these sites.

Quantitative analysis can also help to find the function-related crotonylation sites which are regulated under specific conditions. Sodium crotonate (NACR) can induce protein crotonylation through enzymatic reaction. HDAC1, an endogenously crotonylated protein, can be identified in HeLa cells under conventional cell culture conditions. In addition to HDAC1, after NACR treatment, CBX3, CBX5, MTA2, and Cul4B are also crotonylated, while NACR does not affect the expression of these proteins [49]. In A549 cells treated with SAHA, the proteins with down-regulated Kcr levels were highly enriched in metabolism, while the proteins with up-regulated Kcr level were enriched in biological processes such as protein folding, RNA stability, gene expression, and transcription [21]. It can be anticipated that quantitative proteomics will continue to play an important role in deciphering the cellular role of crotonylation.

### Biological functions of non-histone protein crotonylation

The widespread existence of non-histone protein crotonylation draws attention to the more general pathway control layer mediated by protein crotonylation. Positively charged lysine residues are often involved in protein–protein interaction and protein catalytic activity. Crotonylation neutralizes the positive charge of lysine, thus affecting many aspects of protein function, such as gene transcription, DNA damage response, enzymes regulation, metabolic pathways, cell cycle, and localization of heterochromatin in the cell. Different protein crotonylation could result in different consequences, depending on the functional nature of the proteins.

#### Gene transcription

Kcr is a specific marker of sex-linked genes [73]. Histone crotonylation was first found to be directly related to gene transcription [7]. Histone crotonylation specifically labels enhancers and transcriptional initiation sites of active genes in human somatic genomes and male mouse germ cell genomes, affects the structure of chromatin, and promotes the prolongation of histone substitution in sperm cells [7, 74, 75]. Although the knowledges about the functions of protein crotonylation are largely from studies of histone crotonylation, that of the non-histone crotonylation have been increasingly discovered. Following are two examples about the roles of non-histone crotonylation in gene transcription.

#### P300/CBP

As a typical lysine crotonyltransferase (KCT), P300-mediated crotonylation can promote transcription [31, 61]. In addition, crotonyl-CoA can stimulate transcription more effectively than acetyl-CoA. It has been found that CBP/P300 I1395G mutant (only having KCT activity but not KAT activity) can replace endogenous CBP/P300 KCT to crotonylate promoter and enhance intracellular transcriptional activation, thus improving TGF-β1-induced activation of PAI1 and SMAD7 [32]. Stimulation of macrophages by LPS initiates a transcriptional program that requires P300 recruitment to many sites of the downstream gene.

#### P53

P53 is a tumor suppressor protein which binds to specific DNA sequences and transcriptionally activates target genes to regulate several key cellular processes, including cell cycle control, apoptosis, and DNA repair in response to genotoxic stress [76]. Acetylation of P53 and its role in regulating tumor suppression have been widely explored [77]. The acetylation at multiple lysine residues of P53 by P300/CBP promotes p53 gene transcription and the expression of proapoptotic genes [78]. At the same time, acetylation of the lysine residues of P53 stabilizes P53 by unbinding P53 to its E3 ubiquitin ligase MDM2 [79], while MDM2 in turn recruits HDAC1 to deacetylate P53 and reduce its stability [80]. Large-scale identification of protein crotonylation reveals that P53 can also be crotonylated. However, whether crotonylation of P53 has a lysine residue similar to acetylation and how crotonylation of P53 affects its activity remain to be determined [49].

#### DNA repair

DNA, which carries all the instructions for organisms to survive and reproduce, must remain unharmed in order to replicate accurately before cell division. Therefore, when cells undergo thousands of different types of DNA lesions that endanger the stability of the genome, the lesions must be faithfully repaired with the help of DNA repair proteins. These DNA repair proteins are often mutated in human tumors [81]. In order to cope with a large number of endogenous and exogenous DNA lesions, cells have evolved intertwined but highly orchestrated DNA damage response (DDR) networks, including the signal cascade response that senses DNA lesions and activates downstream cellular pathways to repair damaged DNA [82]. Of note, histone and non-histone PTM play a central role in labeling damaged DNA and stimulating DNA lesion response proteins to recruit and accumulate to DNA breakpoints to promote DNA repair. In this aspect, crotonylation of proteins takes a key role for DNA repair.

As a single-stranded DNA (ssDNA)-binding protein in eukaryotic cells, the replicative protein A (RPA) plays a key role in DNA metabolism in meiosis, such as DNA replication, repair, and homologous recombination (HR). Human RPA is a heterotrimer composed of RPA1, RPA2, and RPA3. Among them, RPA1 is responsible for the binding and interaction of ssDNA and DNA metabolism-related factors [83, 84]. RPA1 interacts with Mre11-Rad50-NBS1 (MRN) complex to promote ssDNA terminal excision during HR, and cooperates with RAD51, BRCA2, and RAD52 to stimulate strand exchange [85, 86]. In addition, RPA1 interacts with WRN and DNA2L to stimulate WRN-mediated double-stranded DNA terminal dissociation and DNA2L-mediated ssDNA degradation [87]. RPA1 is regulated by various PTMs such as acetylation, phosphorylation, ubiquitin, and SUMOylation during DNA metabolism [88–90].

Upon DNA insults induced by camptothecin (CPT), the Kcr level of RPA1 is upregulated significantly. CPT is a recognized drug that induces replication fork collapse, S-phase arrest, and HR in DSB repair of tumor cells. CPT can cancel the reconnection activity of topoisomerase I and produce double-strand break (DSB) [91]. CPT treatment results in a significant increase of Kcr levels in RPA1-K88 and RPA1K379, but only a slight increase in the Kcr level of RPA1-K595. Since both K379 and K595 are located in the DNA binding domain of RPA1, the K379cr and K595cr of RPA1 may be more important to the ssDNA binding ability of RPA1 [52].

**Fig. 3:**
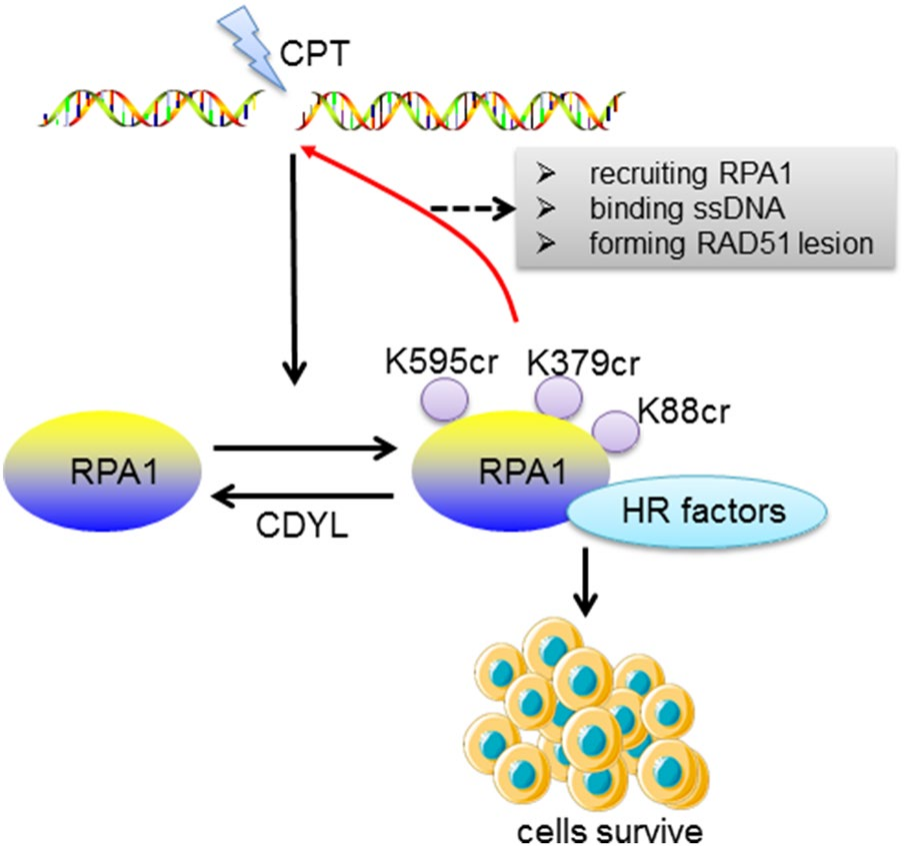
The role of RPA1 Kcr in camptothecin (CPT)-induced double-strand break (DSB) of DNA. RPA1 takes an important role in DNA repair. The Kcr levels at K88, K379, and K595 sites of RPA1 are upregulated by DNA damage but downregulated by CDYL. Sites K379 and K595 locate at the DNA binding domain of RPA1 and may be more important than site K88 for the ssDNA binding ability of RPA1. Crotonylation of RPA1 promotes the binding of RPA1 to ssDNA, recruits RPA1 to the site of DNA damage, enhances the interaction of RPA1 with homologous recombination (HR) factors (including BLM, DNA2L, Mre11, NBS1, and RAD51), and promotes the formation of RAD51 lesions after CPT injury. As a result, the Kcr of RPA1 may make the cells survive and resistant to apoptosis after DNA damage (referenced to: Yu H et al. (2020) Sci Adv 6:eaay4697)

The Kcr of RPA1 plays a key role in homologous recombination-mediated DNA repair. In the process of CPT-induced DSB, the Kcr of RPA1 promotes its ssDNA binding ability, recruits RPA1 at the site of CPT-induced DNA injury, enhances its interaction with HR factors (such as BLM, DNA2L, Mre11, NBS1, and RAD51), facilitates RAD51 foci formation upon CPT insult, and promotes the formation of ssDNA triggered by CPT by interacting with several components of the resection mechanism including MRN complex, BLM, and DNA2L [52]. In HeLa cells treated with CPT, downregulation of RPA1 gene causes a significant increase in apoptosis which can be rescued by the overexpression of wild-type RPA1 rather than mutation of the Kcr sites of RPA1 [52]. It is suggested that the Kcr of RPA1 possibly makes cells survive and resistant to apoptosis under the condition of DNA damage (Fig. 3).

CDYL may regulate RPA1 Kcr dynamics in response to CPT insult. CDYL is required for HR-mediated DNA repair by promoting homology-directed repair of DSBs using “traffic-light reporter” system [92] and coordinating with histone chaperone CAF-1 and DNA helicase minichromosome maintenance (MCM) for proper chromatin structure at damage sites [91]. Although studies have shown that Kcr of RPA1 at K88, K379, and K595 are all negatively regulated by CDYL [52], the possibility cannot be ruled out that RPA1 Kcr may be regulated by other factors, such as crotonyltransferase and/or decrotonylases, which may also contribute to the dynamic changes of RPA1 Kcr after CPT treatment.

#### Enzyme regulation

Large-scale identifications indicate that many enzymes are crotonylated in certain conditions and crotonylation may affect enzyme activities. For example, after treatment with a HDAC inhibitor SAHA in A549 cells, the crotonylation levels of 30 proteins are upregulated at 40 lysine sites, these proteins include aldo–keto reductase, alpha-enolase, acyl-CoA-binding protein, heat shock protein (HSP) 90-alpha, L-lactate dehydrogenase, ribosomal L1 domain-containing protein 1, splicing factor, and 40S ribosomal proteins [21]. However, the crotonylated lysine sites of other enzymes are downregulated, such as K280 on aldehyde dehydrogenase and K419 on very long-chain acyl-CoA synthetase [21]. The activity of a certain enzyme is increased by crotonylation, but not by upregulation of enzyme expression [49]. Expression of HA labeled HDAC1 in HeLa cells is not affected by NaCr or by combined treatment of HDAC inhibitor TSA and nicotinamide (NAM), but NaCr promotes the crotonylation of HDAC1, and the decrotonylase activity of crotonylated HDAC1 on its histone substrate is lower than that of unmodified HDAC1. These studies reveal the importance of crotonylation in the regulation of enzyme activity.

#### Metabolic pathways

Enrichment analyses based on GO annotation, KEGG pathway, and Pfam domain analysis show that crotonylated proteins participate in a variety of important cellular pathways and perform different functions. Crotonylated proteins are involved in many metabolic pathways, including ribosome, spliceosome, proteasome, and Parkinson's disease pathway, and are significantly enriched in ribosomal structure, translation factor activity, and adenyl nucleotide binding in the human lung adenocarcinoma cell line H1299 [17]. Bioinformatics analysis shows that a considerable number of crotonylated non-histone proteins in human specimens are involved in a wide range of metabolic processes, including DNA and RNA metabolism (Table 3). Crotonylated proteins associated with metabolic pathways have a wide range of significance among different species [69]. Crotonylation in papaya is involved in carbon metabolism, antibiotic biosynthesis, amino acid biosynthesis, and glycolysis. In particular, forty crotonylated enzymes have been identified in the amino acid metabolic pathways, suggesting that crotonylation has a potentially conserved function in the regulation of amino acid metabolism in papaya [69].

#### Cell cycle

Crotonylated proteins are enriched in the cell cycle protein network. As the components of histone deacetylase complexes, ubiquitin ligase RNF2 and binding enzymes UBE2E1, NCOR1, and RBBP4 are thought to inhibit histone acetylation and can been crotonylated in the regulation process of chromatin organization and cell cycle [49].

In metazoans, members of the MCM family 2–7 form a hexamer complex at the DNA replication fork, which determines the initiation of DNA replication [93]. After NaCr treatment, the MCM protein in chromatin decreases significantly, and MCM3 is crotonylated [49], which indicates that DNA replication may be affected by protein crotonylation. The combination of these factors may inhibit DNA replication and affect the cell cycle.

#### Localization of heterochromatin

Heterochromatin protein 1α (Hp1α), also known as chromobox homolog 5 (CBX5), is a member of the heterochromatin family and is enriched in heterochromatin via binding with methylated histones [94]. The crotonylation of Hp1α (CBX5) changes its redistribution in the nucleus and reduces the binding to methylated H3K9, the latter is highly enriched in heterochromatin [49]. It is well known that Hp1α acetylation also reduces its binding to methylated H3K9 [95], suggesting that crotonylation of some proteins share similar functional role as acetylation.

### Non-histone protein crotonylation in disease and as potential therapeutic targets

Misregulation of non-histone protein crotonylation is associated with a variety of human diseases, thus crotonylation-related proteins are potential therapeutic targets. Targeted treatments with small inhibitors of HDACs, KATs, and bromine domain proteins have become attractive therapeutic strategies. Although the biological effects of HDACs, KATs, and bromine domain inhibitors are usually related to histone acetylation, these drugs also regulate crotonylation, thus it is possible that non-histone crotonylation is also involved in their cellular effects.

#### Cancer

Cancer-related proteins are affected by Kcr through CBP/P300. By comparing the global quantitative proteomics of p300 knockout cells and wild cells, it is found that the protein biomarkers of cancer in 4.5% of the EDRN database are crotonylated, and 32 Kcr proteins are related to cancer genes, accounting for 5.9% of the total genes of COSMIC cancer gene bank, and 6 target proteins of P300 are identified as cancer gene related proteins [61].

Pairing the tumor specimens with adjacent normal tissues, immunohistochemical staining shows that lysine crotonylation occurs in both cytoplasm and nucleus, and the level of lysine crotonylation is downregulated in liver cancer, gastric cancer, and renal carcinoma, but is upregulated in thyroid, esophagus, colon, pancreas, and lung cancers [96]. Interestingly, in hepatocellular carcinoma, lysine crotonylation level is associated with the stage of tumor lymph node metastasis (TNM).

Knocking down HDAC or adding HDAC inhibitor TSA can increase the level of crotonylation and inhibit the motility and proliferation of hepatocellular carcinoma cells [96], suggesting that HDAC inhibitors may target Kcr. HDAC has been developed to a potential target for oncology therapy. Extensive efforts over the past 20 years have produced dozens of HDAC inhibitors, of which four HDAC inhibitors (voinostat, romidessin, panabinostat, and belinostat) have been clinically approved for the treatment of cutaneous and peripheral T-cell lymphomas as well as multiple myeloma, and has been tested in several other cancers [97].

As a well-known HDAC inhibitor, SAHA can inhibit HDACI, HDAC IIa, and HDAC IIb, and has been approved for the treatment of refractory cutaneous T-cell lymphoma (CTCL) [98]. The therapeutic effect of SAHA on other tumors, such as non-small cell lung cancer (NSCLC) and breast cancer, has also been confirmed [99–101]. In addition, a series of inhibitors of the unique π-π-π domain of readers on the Kcr recognition sites have been developed. For example, XL-13 m, a selective inhibitor of ENL YEATS, can interfere with ENL recruitment on chromatin when it binds to endogenous ENL and cooperatively downregulate oncogenes in acute leukemia with MLL rearrangement [102]. As mentioned above, SAHA can enhance the Kcr of histone and non-histone proteins, but the mechanism of Kcr in SAHA or reader in the treatment of cancer remains to be studied.

#### Major depressive disorder

BTBR T Itpr3tf/J (BTBR) mice have developmental disorders in the central nervous system and show many abnormal neuroanatomical structures. Compared with B6 mice, the global Kcr level of the cerebral cortex of BTBR mice is increased [103], suggesting that the functional relationship between crotonylation and brain diseases is of great significance. About 70 kDa of proteins in mouse brain extract can be recognized by pan Kcr antibodies, indicating the crotonylated non-histone proteins in the brain [104].

### Crosstalk of lysine crotonylation with lysine acetylation

Herein crosstalk means that proteins are modified by multiple PTMs and these PTMs can interact with each other. PTM crosstalk can integrate different signals and greatly improve their regulation potential. Different PTM can lead to competitive crosstalk, fighting for the same lysine residue. Since Kcr and lysine acetylation (Kac) are catalyzed by the same enzymes and are removed by the same enzymes, we give a brief discussion on the crosstalk between Kcr and Kac below.

By comparing the data of crotonylation with that of acetylation on non-histone proteins in A549 cells, it is found that a total of 548 sites are simultaneously modified by both lysine crotonylation and acetylation. After SAHA treatment, 40 sites of 30 non-histone proteins are upregulated at the crotonylation and acetylation levels [21]. This result is consistent with the conclusions of other studies [36, 49], indicating that the two modifications can share a common set of proteins and sites. It is known that the acetyltransferase P300/CBP also catalyzes protein crotonylation, but how essentially the same transferase chooses between Kcr and Kac of the substrate warrants further study.

Whether histone lysine is crotonylated or acetylated depends on the relative concentration of intracellular crotonyl-CoA and acetyl-CoA [40]. In the case of nutritional exhaustion, the level of acetyl-CoA decreases significantly, while the proportion of crotonylated histones may increase to preserve the transcription of key genes during starvation. The fluctuation of protein crotonylation associated with metabolic status may be a normal function of cell physiology and, when destroyed, may lead to disease [105, 106]. Short-chain fatty acid metabolism occurs in intestinal microflora and affects the epigenetic characteristics of multiple proteins [107], including colonic histone Kcr which is dynamically regulated by the cell cycle [104]. When cell energy is exhausted, peroxisomal fatty acid β-oxidation and H3K9 crotonylation increase and is accompanied by a decrease in the levels of ATP and acetyl-CoA, as well as a decrease in the expression of ribosomal biogenic genes [108]. In this metabolic state, the crotonyl reader Taf14 YEATS domain inhibits the expression of growth-promoting genes to adapt to the low-energy state of cells [109]. We speculate that crotonylation of non-histone protein is also regulated by the relative concentrations of crotonyl-CoA and acetyl-CoA to adapt to the changes of cell energy environment.

## Future perspectives

MS-based quantitative proteomics defines an initial reference framework for comprehensive Kcr studies and helps to our understanding on non-histone protein crotonylation. The latest developments in high-resolution nuclear magnetic resonance (NMR), genome engineering, novel bioinformatics tools, and selective chemical probe development provide exciting opportunities for system-wide and detailed mechanism studies to meet future challenges [110–114].

Although crotonylation and acetylation share certain enzymes, the properties of many newly discovered enzymes that regulate non-histone protein crotonylation are still unknown. Whether the enzymes catalyze in a site-selective manner or the enzymes shuttle in different cell compartments are interesting issues to be studied in the future.

Non-histone protein crotonylation does regulate many metabolic enzymes, but the dynamic regulation mechanism of the modification sites has not been deeply studied. Future work may find more evidence on how non-histone crotonylation affects protein and cell function. Crotonylation has been proved related with many human diseases, which may open an attractive field for medical intervention. We further expect that non-histone Kcr may be a contributor to disease development and a therapeutic target. Similar to other rapidly developing research fields, studies on non-histone protein crotonylation in the near future may face both challenges and exciting discoveries.

## Data Availability

Not applicable.

## Abbreviations

ACSS2: Acyl-CoA synthetase short chain family member 2
AF9: ALL1 fused gene from chromosome 9
CBX5: Chromobox homolog 5
CDYL: Chromodomain Y-like
CoA: Coenzyme A
CPT: Camptothecin
CTCL: Cutaneous T-cell lymphoma
DDR: DNA damage response
DDX5: DEAD-box helicase 5
DPF: Double plant homeodomain finger
DSB: Double-strand break
GCN5: General control nonrepressed-protein 5
GNAT: GCN5-related N-acetyltransferase
HDAC: Histone deacetylase
hMOF: Human males absent on the first
Hp1α: Heterochromatin protein 1α
HR: Homologous recombination
HSP: Heat shock protein
IgAN: Immunoglobulin A nephropathy
InsP: Inositol phosphates
IR: Ionizing radiation
Kac: Lysine acetylation
Kcr: Lysine crotonylation
KCT: Lysine crotonyltransferase
KDCR: Lysine decrotonylase
LC-MS/MS: Liquid chromatography coupled to tandem MS
LTR: Long-terminal repeat
MCM: Minichromosome maintenance
MOZ: Monocytic leukemia zinc-finger protein
mPFC: Medial prefrontal cortex
MS: Mass spectrometry
MYST: Moz Ybf2 Sas2 and Tip60
NaCr: Sodium crotonate
NMR: Nuclear magnetic resonance
NPM1: Nucleophosmin-1
NSCLC: Non-small cell lung cancer
PAT: Patulin
PBMC: Peripheral blood mononuclear cells
P300/CBP: P300/CREB-binding protein
PCAF: P300/CBP-associated factor
PHD: Plant homeodomain finger
PTM: Post-translational modification
RPA: Replicative protein A
SAHA: Suberoylanilide hydroxamic acid
SCFA: Short-chain fatty acid
SIRT: Sirtuin family deacetylase
ssDNA: Single-stranded DNA
TAF1: TATA binding protein-associated factor-1
Taf14: TATA binding protein-associated factor-14
*T. gondii*: Toxoplasma gondii parasites
TNM: Tumor lymph node metastasis
TSA: Trichostatin A

## References

[1] Wang ZA, Kurra Y, Wang X, Zeng Y, Lee YJ, Sharma V (2017). A versatile approach for site-specific lysine acylation in proteins. Angew Chem Int Ed Engl.

[2] Yan SB, Wold F (1984). Neoglycoproteins: in vitro introduction of glycosyl units at glutamines in beta-casein using transglutaminase. Biochemistry.

[3] Garrity J, Gardner JG, Hawse W, Wolberger C, Escalante-Semerena JC (2007). N-lysine propionylation controls the activity of propionyl-CoA synthetase. J Biol Chem.

[4] van Slyke DD, Sinex FM (1958). The course of hydroxylation of lysine to form hydroxylysine in collagen. J Biol Chem.

[5] Sutendra G, Kinnaird A, Dromparis P, Paulin R, Stenson TH, Haromy A (2014). A nuclear pyruvate dehydrogenase complex is important for the generation of acetyl-CoA and histone acetylation. Cell.

[6] Das D, Bandyopadhyay D, Banerjee RK (1998). Oxidative inactivation of gastric peroxidase by site-specific generation of hydroxyl radical and its role in stress-induced gastric ulceration. Free Radic Biol Med.

[7] Tan M, Luo H, Lee S, Jin F, Yang JS, Montellier E (2011). Identification of 67 histone marks and histone lysine crotonylation as a new type of histone modification. Cell.

[8] Hochstrasser M (2009). Origin and function of ubiquitin-like proteins. Nature.

[9] Wisniewski JR, Zougman A, Mann M (2008). Nepsilon-formylation of lysine is a widespread post-translational modification of nuclear proteins occurring at residues involved in regulation of chromatin function. Nucleic Acids Res.

[10] Peng C, Lu Z, Xie Z, Cheng Z, Chen Y, Tan M (2011). The first identification of lysine malonylation substrates and its regulatory enzyme. Mol Cell Proteomics.

[11] Zhang Z, Tan M, Xie Z, Dai L, Chen Y, Zhao Y (2011). Identification of lysine succinylation as a new post-translational modification. Nat Chem Biol.

[12] Lan F, Shi Y (2009). Epigenetic regulation: methylation of histone and non-histone proteins. Sci China C Life Sci.

[13] Ruiz-Andres O, Sanchez-Niño MD, Cannata-Ortiz P, Ruiz-Ortega M, Egido J, Ortiz A (2016). Histone lysine crotonylation during acute kidney injury in mice. Dis Model Mech.

[14] Liu Y, Li M, Fan M, Song Y, Yu H, Zhi X (2019). Chromodomain y-like protein-mediated histone crotonylation regulates stress-induced depressive behaviors. Biol Psychiatry.

[15] Jiang G, Nguyen D, Archin NM, Yukl SA, Méndez-Lagares G, Tang Y (2018). HIV latency is reversed by ACSS2-driven histone crotonylation. J Clin Invest.

[16] Berger K, Moeller MJ (2014). Mechanisms of epithelial repair and regeneration after acute kidney injury. Semin Nephrol.

[17] Xu W, Wan J, Zhan J, Li X, He H, Shi Z (2017). Global profiling of crotonylation on non-histone proteins. Cell Res.

[18] Kwon OK, Kim SJ, Lee S (2018). First profiling of lysine crotonylation of myofilament proteins and ribosomal proteins in zebrafish embryos. Sci Rep.

[19] Wan J, Liu H, Chu J, Zhang H (2019). Functions and mechanisms of lysine crotonylation. J Cell Mol Med.

[20] Liu JF, Wu SF, Liu S, Sun X, Wang XM, Xu P (2020). Global lysine crotonylation profiling of mouse liver. Proteomics.

[21] Wu Q, Li W, Wang C, Fan P, Cao L, Wu Z (2017). Ultradeep Lysine Crotonylome Reveals the Crotonylation Enhancement on Both Histones and Nonhistone Proteins by SAHA Treatment. J Proteome Res.

[22] Chen Y, Chen W, Cobb MH, Zhao Y (2009). PTMap–a sequence alignment software for unrestricted, accurate, and full-spectrum identification of post-translational modification sites. Proc Natl Acad Sci U S A.

[23] Kotliński M, Rutowicz K, Kniżewski Ł, Palusiński A, Olędzki J, Fogtman A (2016). Histone H1 variants in arabidopsis are subject to numerous post-translational modifications, both conserved and previously unknown in histones, suggesting complex functions of H1 in plants. PLoS ONE.

[24] Montellier E, Boussouar F, Rousseaux S, Zhang K, Buchou T, Fenaille F (2013). Chromatin-to-nucleoprotamine transition is controlled by the histone H2B variant TH2B. Genes Dev.

[25] Sun H, Liu X, Li F, Li W, Zhang J, Xiao Z (2017). First comprehensive proteome analysis of lysine crotonylation in seedling leaves of *Nicotiana tabacum*. Sci Rep.

[26] Tweedie-Cullen RY, Brunner AM, Grossmann J, Mohanna S, Sichau D, Nanni P (2012). Identification of combinatorial patterns of post-translational modifications on individual histones in the mouse brain. PLoS ONE.

[27] Tanner KG, Landry J, Sternglanz R, Denu JM (2000). Silent information regulator 2 family of NAD- dependent histone/protein deacetylases generates a unique product, 1-O-acetyl-ADP-ribose. Proc Natl Acad Sci U S A.

[28] Tanny JC, Moazed D (2001). Coupling of histone deacetylation to NAD breakdown by the yeast silencing protein Sir2: Evidence for acetyl transfer from substrate to an NAD breakdown product. Proc Natl Acad Sci U S A.

[29] Li Y, Sabari BR, Panchenko T, Wen H, Zhao D, Guan H (2016). Molecular coupling of histone crotonylation and active transcription by AF9 YEATS domain. Mol Cell.

[30] Zhao S, Zhang X, Li H (2018). Beyond histone acetylation-writing and erasing histone acylations. Curr Opin Struct Biol.

[31] Sabari BR, Tang Z, Huang H, Yong-Gonzalez V, Molina H, Kong HE (2018). Intracellular Crotonyl-CoA stimulates transcription through p300-catalyzed histone crotonylation. Mol Cell.

[32] Liu X, Wei W, Liu Y, Yang X, Wu J, Zhang Y (2017). MOF as an evolutionarily conserved histone crotonyltransferase and transcriptional activation by histone acetyltransferase-deficient and crotonyltransferase-competent CBP/p300. Cell Discov.

[33] Kollenstart L, de Groot A, Janssen G, Cheng X, Vreeken K, Martino F (2019). Gcn5 and Esa1 function as histone crotonyltransferases to regulate crotonylation-dependent transcription. J Biol Chem.

[34] Zhao D, Guan H, Zhao S, Mi W, Wen H, Li Y (2016). YEATS2 is a selective histone crotonylation reader. Cell Res.

[35] Andrews FH, Shinsky SA, Shanle EK, Bridgers JB, Gest A, Tsun IK (2016). The Taf14 YEATS domain is a reader of histone crotonylation. Nat Chem Biol.

[36] Wei W, Liu X, Chen J, Gao S, Lu L, Zhang H (2017). Class I histone deacetylases are major histone decrotonylases: evidence for critical and broad function of histone crotonylation in transcription. Cell Res.

[37] Feldman JL, Baeza J, Denu JM (2013). Activation of the protein deacetylase SIRT6 by long-chain fatty acids and widespread deacylation by mammalian sirtuins. J Biol Chem.

[38] Bao X, Wang Y, Li X, Li XM, Liu Z, Yang T (2014). Identification of 'erasers' for lysine crotonylated histone marks using a chemical proteomics approach. Elife.

[39] Martinez-Moreno JM, Fontecha-Barriuso M, Martín-Sánchez D, Sánchez-Niño MD, Ruiz-Ortega M, Sanz AB (2020). The contribution of histone crotonylation to tissue health and disease: focus on kidney health. Front Pharmacol.

[40] Sabari BR, Tang Z, Huang H, Yong-Gonzalez V, Molina H, Kong HE (2015). Intracellular crotonyl-CoA stimulates transcription through p300-catalyzed histone crotonylation. Mol Cell.

[41] Haberland M, Montgomery RL, Olson EN (2009). The many roles of histone deacetylases in development and physiology: implications for disease and therapy. Nat Rev Genet.

[42] Houtkooper RH, Pirinen E, Auwerx J (2012). Sirtuins as regulators of metabolism and healthspan. Nat Rev Mol Cell Biol.

[43] Sauve AA, Wolberger C, Schramm VL, Boeke JD (2006). The biochemistry of sirtuins. Annu Rev Biochem.

[44] Flynn EM, Huang OW, Poy F, Oppikofer M, Bellon SF, Tang Y (2015). A subset of human bromodomains recognizes butyryllysine and crotonyllysine histone peptide modifications. Structure.

[45] Xiong X, Panchenko T, Yang S, Zhao S, Yan P, Zhang W (2016). Selective recognition of histone crotonylation by double PHD fingers of MOZ and DPF2. Nat Chem Biol.

[46] Zhang Q, Zeng L, Zhao C, Ju Y, Konuma T, Zhou MM (2016). Structural insights into histone crotonyl-lysine recognition by the AF9 YEATS domain. Structure.

[47] Stilling RM, van de Wouw M, Clarke G, Stanton C, Dinan TG, Cryan JF (2016). The neuropharmacology of butyrate: the bread and butter of the microbiota-gut-brain axis. Neurochem Int.

[48] Lin H, Su X, He B (2012). Protein lysine acylation and cysteine succination by intermediates of energy metabolism. ACS Chem Biol.

[49] Wei W, Mao A, Tang B, Zeng Q, Gao S, Liu X (2017). Large-scale identification of protein crotonylation reveals its role in multiple cellular functions. J Proteome Res.

[50] Caron C, Pivot-Pajot C, van Grunsven LA, Col E, Lestrat C, Rousseaux S (2003). Cdyl: a new transcriptional co-repressor. EMBO Rep.

[51] Liu S, Yu H, Liu Y, Liu X, Zhang Y, Bu C (2017). Chromodomain protein CDYL acts as a Crotonyl-CoA hydratase to regulate histone crotonylation and spermatogenesis. Mol Cell.

[52] Yu H, Bu C, Liu Y, Gong T, Liu X, Liu S (2020). Global crotonylome reveals CDYL-regulated RPA1 crotonylation in homologous recombination-mediated DNA repair. Sci Adv.

[53] Ong SE, Blagoev B, Kratchmarova I, Kristensen DB, Steen H, Pandey A (2002). Stable isotope labeling by amino acids in cell culture, SILAC, as a simple and accurate approach to expression proteomics. Mol Cell Proteomics.

[54] Yang Q, Li Y, Apaliya MT, Zheng X, Serwah B, Zhang X (2018). The Response of *Rhodotorula mucilaginosa* to patulin based on lysine crotonylation. Front Microbiol.

[55] Evans C, Noirel J, Ow SY, Salim M, Pereira-Medrano AG, Couto N (2012). An insight into iTRAQ: where do we stand now. Anal Bioanal Chem.

[56] Chen W, Tang D, Xu Y, Zou Y, Sui W, Dai Y (2018). Comprehensive analysis of lysine crotonylation in proteome of maintenance hemodialysis patients. Medicine.

[57] Cox J, Hein MY, Luber CA, Paron I, Nagaraj N, Mann M (2014). Accurate proteome-wide label-free quantification by delayed normalization and maximal peptide ratio extraction, termed MaxLFQ. Mol Cell Proteomics.

[58] Zhao L, Cong X, Zhai L, Hu H, Xu JY, Zhao W (2020). Comparative evaluation of label-free quantification strategies. J Proteomics.

[59] Lv H, Dao FY, Guan ZX, Yang H, Li YW, Lin H (2020). Deep-Kcr: accurate detection of lysine crotonylation sites using deep learning method. Brief Bioinform.

[60] Sun J, Qiu C, Qian W, Wang Y, Sun L, Li Y (2019). Ammonium triggered the response mechanism of lysine crotonylome in tea plants. BMC Genomics.

[61] Huang H, Wang DL, Zhao Y (2018). Quantitative crotonylome analysis expands the roles of p300 in the regulation of lysine crotonylation pathway. Proteomics.

[62] Michalski A, Cox J, Mann M (2011). More than 100,000 detectable peptide species elute in single shotgun proteomics runs but the majority is inaccessible to data-dependent LC-MS/MS. J Proteome Res.

[63] Altelaar AF, Munoz J, Heck AJ (2013). Next-generation proteomics: towards an integrative view of proteome dynamics. Nat Rev Genet.

[64] Aebersold R, Mann M (2016). Mass-spectrometric exploration of proteome structure and function. Nature.

[65] Eliuk S, Makarov A (2015). Evolution of orbitrap mass spectrometry instrumentation. Annu Rev Anal Chem (Palo Alto Calif).

[66] Meier F, Beck S, Grassl N, Lubeck M, Park MA, Raether O (2015). Parallel accumulation-serial fragmentation (PASEF): multiplying sequencing speed and sensitivity by synchronized scans in a trapped ion mobility device. J Proteome Res.

[67] Meier F, Brunner AD, Koch S, Koch H, Lubeck M, Krause M (2018). Online parallel accumulation-serial fragmentation (PASEF) with a novel trapped ion mobility mass spectrometer. Mol Cell Proteomics.

[68] Lin H, Tang D, Xu Y, Zhang R, Ou M, Zheng F (2020). Quantitative analysis of protein crotonylation identifies its association with immunoglobulin A nephropathy. Mol Med Rep.

[69] Liu K, Yuan C, Li H, Chen K, Lu L, Shen C (2018). A qualitative proteome-wide lysine crotonylation profiling of papaya (*Carica papaya* L.). Sci Rep.

[70] Liu S, Xue C, Fang Y, Chen G, Peng X, Zhou Y (2018). Global involvement of lysine crotonylation in protein modification and transcription regulation in rice. Mol Cell Proteomics.

[71] Yin D, Jiang N, Zhang Y, Wang D, Sang X, Feng Y (2019). Global lysine crotonylation and 2-hydroxyisobutyrylation in phenotypically different *Toxoplasma gondii* parasites. Mol Cell Proteomics.

[72] Kemp BE, Pearson RB (1990). Protein kinase recognition sequence motifs. Trends Biochem Sci.

[73] Montellier E, Rousseaux S, Zhao Y, Khochbin S (2012). Histone crotonylation specifically marks the haploid male germ cell gene expression program: post-meiotic male-specific gene expression. BioEssays.

[74] Kolthur-Seetharam U, Martianov I, Davidson I (2008). Specialization of the general transcriptional machinery in male germ cells. Cell Cycle.

[75] Sassone-Corsi P (2002). Unique chromatin remodeling and transcriptional regulation in spermatogenesis. Science.

[76] Vogelstein B, Kinzler KW (1992). p53 function and dysfunction. Cell.

[77] Dai C, Gu W (2010). p53 post-translational modification: deregulated in tumorigenesis. Trends Mol Med.

[78] Brooks CL, Gu W (2011). The impact of acetylation and deacetylation on the p53 pathway. Protein Cell.

[79] Reed SM, Hagen J, Tompkins VS, Thies K, Quelle FW, Quelle DE (2014). Nuclear interactor of ARF and Mdm2 regulates multiple pathways to activate p53. Cell Cycle.

[80] Ito A, Kawaguchi Y, Lai CH, Kovacs JJ, Higashimoto Y, Appella E (2002). MDM2-HDAC1-mediated deacetylation of p53 is required for its degradation. EMBO J.

[81] Chae YK, Anker JF, Carneiro BA, Chandra S, Kaplan J, Kalyan A (2016). Genomic landscape of DNA repair genes in cancer. Oncotarget.

[82] Ciccia A, Elledge SJ (2010). The DNA damage response: making it safe to play with knives. Mol Cell.

[83] Fanning E, Klimovich V, Nager AR (2006). A dynamic model for replication protein A (RPA) function in DNA processing pathways. Nucleic Acids Res.

[84] Toledo LI, Altmeyer M, Rask MB, Lukas C, Larsen DH, Povlsen LK (2013). ATR prohibits replication catastrophe by preventing global exhaustion of RPA. Cell.

[85] Prakash R, Zhang Y, Feng W, Jasin M (2015). Homologous recombination and human health: the roles of BRCA1, BRCA2, and associated proteins. Cold Spring Harb Perspect Biol.

[86] Syed A, Tainer JA (2018). The MRE11-RAD50-NBS1 complex conducts the orchestration of damage signaling and outcomes to stress in DNA replication and repair. Annu Rev Biochem.

[87] Tammaro M, Liao S, McCane J, Yan H (2015). The N-terminus of RPA large subunit and its spatial position are important for the 5'->3' resection of DNA double-strand breaks. Nucleic Acids Res.

[88] Dou H, Huang C, Singh M, Carpenter PB, Yeh ET (2010). Regulation of DNA repair through deSUMOylation and SUMOylation of replication protein A complex. Mol Cell.

[89] Elia AE, Wang DC, Willis NA, Boardman AP, Hajdu I, Adeyemi RO (2015). RFWD3-dependent ubiquitination of rpa regulates repair at stalled replication forks. Mol Cell.

[90] He H, Wang J, Liu T (2017). UV-induced RPA1 acetylation promotes nucleotide excision repair. Cell Rep.

[91] Liu Y, Liu S, Yuan S, Yu H, Zhang Y, Yang X (2017). Chromodomain protein CDYL is required for transmission/restoration of repressive histone marks. J Mol Cell Biol.

[92] Abu-Zhayia ER, Awwad SW, Ben-Oz BM, Khoury-Haddad H, Ayoub N (2018). CDYL1 fosters double-strand break-induced transcription silencing and promotes homology-directed repair. J Mol Cell Biol.

[93] Lei M (2005). The MCM complex: its role in DNA replication and implications for cancer therapy. Curr Cancer Drug Targets.

[94] Verschure PJ, van der Kraan I, de Leeuw W, van der Vlag J, Carpenter AE, Belmont AS (2005). In vivo HP1 targeting causes large-scale chromatin condensation and enhanced histone lysine methylation. Mol Cell Biol.

[95] Qiu Y, Zhao Y, Becker M, John S, Parekh BS, Huang S (2006). HDAC1 acetylation is linked to progressive modulation of steroid receptor-induced gene transcription. Mol Cell.

[96] Wan J, Liu H, Ming L (2019). Lysine crotonylation is involved in hepatocellular carcinoma progression. Biomed Pharmacother.

[97] Pant K, Peixoto E, Richard S, Gradilone SA (2020). Role of histone deacetylases in carcinogenesis: potential role in cholangiocarcinoma. Cells.

[98] Olsen EA, Kim YH, Kuzel TM, Pacheco TR, Foss FM, Parker S (2007). Phase IIb multicenter trial of vorinostat in patients with persistent, progressive, or treatment refractory cutaneous T-cell lymphoma. J Clin Oncol.

[99] Chen MY, Liao WS, Lu Z, Bornmann WG, Hennessey V, Washington MN (2011). Decitabine and suberoylanilide hydroxamic acid (SAHA) inhibit growth of ovarian cancer cell lines and xenografts while inducing expression of imprinted tumor suppressor genes, apoptosis, G2/M arrest, and autophagy. Cancer.

[100] Konstantinopoulos PA, Wilson AJ, Saskowski J, Wass E, Khabele D (2014). Suberoylanilide hydroxamic acid (SAHA) enhances olaparib activity by targeting homologous recombination DNA repair in ovarian cancer. Gynecol Oncol.

[101] Munster PN, Troso-Sandoval T, Rosen N, Rifkind R, Marks PA, Richon VM (2001). The histone deacetylase inhibitor suberoylanilide hydroxamic acid induces differentiation of human breast cancer cells. Cancer Res.

[102] Li X, Li XM, Jiang Y, Liu Z, Cui Y, Fung KY (2018). Structure-guided development of YEATS domain inhibitors by targeting π-π-π stacking. Nat Chem Biol.

[103] Wang M, Chang Q, Yang H, Liu Y, Wang C, Hu F (2019). Elevated lysine crotonylation and succinylation in the brains of BTBR mice. Int J Dev Neurosci.

[104] Fellows R, Denizot J, Stellato C, Cuomo A, Jain P, Stoyanova E (2018). Microbiota derived short chain fatty acids promote histone crotonylation in the colon through histone deacetylases. Nat Commun.

[105] Koh A, De Vadder F, Kovatcheva-Datchary P, Bäckhed F (2016). From dietary fiber to host physiology: short-chain fatty acids as key bacterial metabolites. Cell.

[106] Nath A, Chan C (2016). Genetic alterations in fatty acid transport and metabolism genes are associated with metastatic progression and poor prognosis of human cancers. Sci Rep.

[107] Donohoe DR, Garge N, Zhang X, Sun W, O'Connell TM, Bunger MK (2011). The microbiome and butyrate regulate energy metabolism and autophagy in the mammalian colon. Cell Metab.

[108] Cai L, Sutter BM, Li B, Tu BP (2011). Acetyl-CoA induces cell growth and proliferation by promoting the acetylation of histones at growth genes. Mol Cell.

[109] Gowans GJ, Bridgers JB, Zhang J, Dronamraju R, Burnetti A, King DA (2019). Recognition of Histone Crotonylation by Taf14 Links Metabolic State to Gene Expression. Mol Cell.

[110] Ju Z, He JJ (2017). Prediction of lysine crotonylation sites by incorporating the composition of k-spaced amino acid pairs into Chou's general PseAAC. J Mol Graph Model.

[111] Qiu WR, Sun BQ, Tang H, Huang J, Lin H (2017). Identify and analysis crotonylation sites in histone by using support vector machines. Artif Intell Med.

[112] Qiu WR, Sun BQ, Xiao X, Xu ZC, Chou KC (2016). iPTM-mLys: identifying multiple lysine PTM sites and their different types. Bioinformatics.

[113] Theillet FX, Smet-Nocca C, Liokatis S, Thongwichian R, Kosten J, Yoon MK (2012). Cell signaling, post-translational protein modifications and NMR spectroscopy. J Biomol NMR.

[114] Xie X, Li XM, Qin F, Lin J, Zhang G, Zhao J (2017). Genetically encoded photoaffinity histone marks. J Am Chem Soc.

